# Editorial for the Special Issue “Hot Topics and Innovations in Reconstructive and Cosmetic Surgery/Aesthetic Medicine”

**DOI:** 10.3390/medicina61081480

**Published:** 2025-08-18

**Authors:** Luigi Losco

**Affiliations:** Department of Medicine, Surgery and Dentistry, University of Salerno, 84081 Baronissi, Salerno, Italy; llosco@unisa.it

Innovations and emerging trends in Plastic Reconstructive and Aesthetic Surgery—as well as in Aesthetic Medicine—are continuously expanding the frontiers of modern clinical practice. From cutting-edge microsurgical and supermicrosurgical techniques to advancements in non-invasive procedures, regenerative therapies, and the integration of artificial intelligence, these fields are evolving rapidly to enhance both functional restoration and aesthetic enhancement with greater precision, reliability, and safety [1,2,3,4,5]. This Special Issue features five original contributions and a comprehensive review. While these works are indeed heterogeneous, it is relevant to highlight that they collectively represent the wide array of medical and surgical approaches available to our patients, addressing serious health concerns, quality of life issues, or aesthetic considerations. On the other hand, a common thread can be observed, namely, the way recent developments are driving progress, improving patient outcomes, and redefining standards in both surgical and non-surgical reconstructive and aesthetic care.

The prospective, double-blind study by Di Guardo et al. [6] validated the efficacy of the topical product containing hyper concentrated sodium chloride versus a placebo in the treatment of cellulite in female patients with grade II or III cellulite in the thighs and buttocks, according to the Nurnberger–Muller classification [7]. The study primarily focused on objective measurements such as thigh circumference, ultrasound evaluations, and standardised photographic images.

A comprehensive review by Lippi et al. [8] thoroughly depicted the wide range of rehabilitative techniques and interventions commonly employed in rehabilitation settings with a primary emphasis on their capacity to enhance aesthetics, quality of life, and psychosocial wellbeing among individuals facing disabilities [9]. From intricate reconstructive efforts post-trauma or post-surgery to the targeted application of injection therapies and physical modalities, the integration of aesthetic approaches within the field of rehabilitation underscores a synergistic multidisciplinary approach to patient-centric care [10,11].

A retrospective study by Breidung et al. [12] highlighted the challenges in performing chest wall defect reconstruction. Reconstruction is indeed critical to the patient’s quality of life and overall wellbeing: it enables the patient’s respiratory function, protects the lungs, and stabilises the shoulder girdle while mitigating the significant risk of complications such as lung herniation and respiratory compromise [13,14,15].

The authors reported a lower rate of major complications with free flap surgery compared to pedicled flaps, although further studies were recommended to confirm these findings. Moreover, their study emphasised the ongoing need for research to refine surgical techniques and improve patient care in this challenging field. It also highlighted the importance of a multidisciplinary approach and preoperative discussion with patients to optimise their outcomes.

The development of improved and innovative strategies for wound healing holds significant global importance within the healthcare sector both for the patient and for the social costs. In addition to conventional therapies, such as debridement and routine dressing change, more advanced “regenerative” approaches have emerged with the aim to enhance tissue regeneration and promote more effective and timelier wound repair. These include the use of bioactive biomaterial matrices and stem cell-based therapies [16,17,18]. An experimental study by Elia et al. [19] aimed to contribute to research in the field of wound healing by helping to identify the most appropriate treatment strategy. The authors compared different conventional and innovative regenerative approaches to enhance wound healing in a sheep surgical acute wound model; the outcome evaluation was based on clinical and histopathological analyses. The application of a dermal matrix or the use of the micronized dermis system did not enhance the healing of acute, full-thickness trunk wounds in sheep compared to conventional treatments or even compared to non-topical disinfectant application. However, both approaches did appear to improve the phases of wound healing, contributing to the exudate control, promoting angiogenesis, and, in the end, attenuating scar formation. The study calls for further investigations.

Grussu et al. [20] described a unique case report involving the complementary use of Integra DRT and MicroMatrix for the reconstruction of a full-thickness subtotal scalp defect following a dog bite. The complex wound was closed after a multistage application of regenerative matrices and a split-thickness skin graft.

Finally, Gomez-Cabello et al. [21] provided valuable insights into the current state of two readily available learning language models (LLMs), namely ChatGPT-4 and Gemini. In this study, the authors aimed to evaluate and compare the current state of the two most common and readily available LLMs in providing intraoperative decision support in plastic and reconstructive surgery procedures. Both models demonstrated adequate knowledge for supporting surgeons during operative procedures; however, their performance varied across the different surgical scenarios, with neither model consistently delivering completely correct or relevant information [22]. This variability highlights the need for further development and optimisation to ensure their reliability and precision in the intraoperative setting.

In conclusion, the innovative spirit of plastic, reconstructive, and regenerative surgery—together with aesthetic medicine—is reshaping modern healthcare. As research and technology progress hand in hand, the commitment to improving both function and form remains at the heart of this dynamic and inspiring discipline.
